# Adaptive Aquila Optimizer with Explainable Artificial Intelligence-Enabled Cancer Diagnosis on Medical Imaging

**DOI:** 10.3390/cancers15051492

**Published:** 2023-02-27

**Authors:** Salem Alkhalaf, Fahad Alturise, Adel Aboud Bahaddad, Bushra M. Elamin Elnaim, Samah Shabana, Sayed Abdel-Khalek, Romany F. Mansour

**Affiliations:** 1 Department of Computer, College of Science and Arts in Ar Rass, Qassim University, Ar Rass 58892, Saudi Arabia; 2 Department of Information Systems, Faculty of Computing and Information Technology, King Abdulaziz University, Jeddah 21589, Saudi Arabia; 3 Department of Computer Science, College of Science and Humanities in Al-Sulail, Prince Sattam Bin Abdulaziz University, Al-Kharj 16278, Saudi Arabia; 4 Pharmacognosy Department, Faculty of Pharmaceutical Sciences and Drug Manufacturing, Misr University for Science and Technology (MUST), Giza 3236101, Egypt; 5 Department of Mathematics, College of Science, Taif University, Taif 21944, Saudi Arabia; 6 Department of Mathematics, Faculty of Science, New Valley University, El-Kharga 1064188, Egypt

**Keywords:** cancer diagnosis, explainable artificial intelligence, ensemble learning, Adaptive Aquila Optimizer, deep learning

## Abstract

**Simple Summary:**

For automated cancer diagnosis on medical imaging, explainable artificial intelligence technology uses advanced image analysis methods like deep learning to make a diagnosis and analyze medical images, as well as provide a clear explanation for how it arrived at its diagnosis. The objective of XAI is to provide patients and doctors with a better understanding of the system’s decision-making process and to increase transparency and trust in the diagnosis method. The manual classification of cancer using medical images is a tedious and tiresome process, which necessitates the design of automated tools for the decision-making process. In this study, we explored the significant application of explainable artificial intelligence and an ensemble of deep-learning models for automated cancer diagnosis. To demonstrate the enhanced performance of the proposed model, a widespread comparison study is made with recent models, and the results exhibit the significance of the proposed model on benchmark test images. Therefore, the proposed model has the potential as an automated, accurate, and rapid tool for supporting the detection and classification process of cancer.

**Abstract:**

Explainable Artificial Intelligence (XAI) is a branch of AI that mainly focuses on developing systems that provide understandable and clear explanations for their decisions. In the context of cancer diagnoses on medical imaging, an XAI technology uses advanced image analysis methods like deep learning (DL) to make a diagnosis and analyze medical images, as well as provide a clear explanation for how it arrived at its diagnoses. This includes highlighting specific areas of the image that the system recognized as indicative of cancer while also providing data on the fundamental AI algorithm and decision-making process used. The objective of XAI is to provide patients and doctors with a better understanding of the system’s decision-making process and to increase transparency and trust in the diagnosis method. Therefore, this study develops an Adaptive Aquila Optimizer with Explainable Artificial Intelligence Enabled Cancer Diagnosis (AAOXAI-CD) technique on Medical Imaging. The proposed AAOXAI-CD technique intends to accomplish the effectual colorectal and osteosarcoma cancer classification process. To achieve this, the AAOXAI-CD technique initially employs the Faster SqueezeNet model for feature vector generation. As well, the hyperparameter tuning of the Faster SqueezeNet model takes place with the use of the AAO algorithm. For cancer classification, the majority weighted voting ensemble model with three DL classifiers, namely recurrent neural network (RNN), gated recurrent unit (GRU), and bidirectional long short-term memory (BiLSTM). Furthermore, the AAOXAI-CD technique combines the XAI approach LIME for better understanding and explainability of the black-box method for accurate cancer detection. The simulation evaluation of the AAOXAI-CD methodology can be tested on medical cancer imaging databases, and the outcomes ensured the auspicious outcome of the AAOXAI-CD methodology than other current approaches.

## 1. Introduction

Diagnosis of cancer is an indispensable problem in the medical sector. Initial identification of cancer is vital for better chances of treatment and the best course of action [1]. Therefore, cancer can be considered as one major topic where numerous authors carried out various research to attain higher performance in treatment prevention and diagnosis. Initial identification of tumors can increase treatment options and chances of survival of patients. Medical images like Magnetic Resonance Imaging, mammograms, microscopic images, and ultrasound were the typical technique for diagnosing cancer [2].

**Figure 1:**
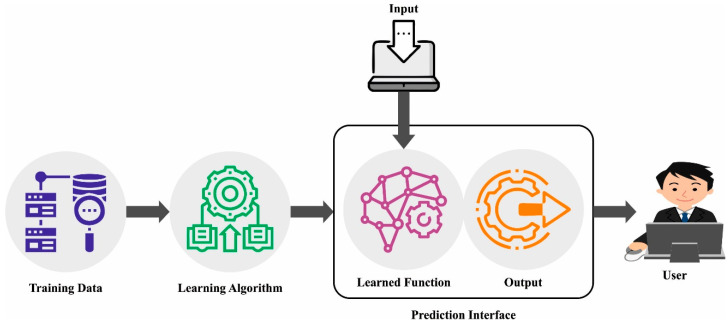
Structure of XAI.

In recent times, computer-aided diagnosis (CAD) mechanism was utilized to help doctors in diagnosing tumors so that the accuracy level of diagnosis gets enhanced. CAD helps in reducing missed cancer lesions because of medical practitioner fatigue, minimizing data overloading and work pressure, and reducing the variability of intra-and-inter readers of imageries [3]. Problems like technical reasons are relevant to imaging quality, and errors caused by humans have augmented the misdiagnosis of breast cancer in the interpretation of radiologists. To solve these limitations, CAD mechanisms were advanced to automate breast cancer diagnosis and categorize malignant and benign lesions [4]. The CAD mechanism enhances the performance of radiologists in discriminating and finding abnormal and normal tissues. Such a process can be executed only as a double reader, but decisions are made by radiologists [5]. Figure 1 represents the structure of explainable artificial intelligence.

Recent advancements in the resolution of medical imaging modalities have enhanced diagnostic accuracy [6]. Effective use of imaging data for enhancing the diagnosis becomes significant. Currently, computer-aided diagnosis systems (CAD) have advanced a novel context in radiology to make use of data that should be implemented in the diagnosis of different diseases and different imaging modalities [7,8,9,10]. The efficacy of radiologists’ analysis can be enhanced in the context of consistency and accuracy in diagnosis or detection, while production can be enhanced by minimizing the hours needed to read the imageries. The results can be extracted through several methods in computer vision (CV) for presenting certain important variables like the likelihood of malignancy and the location of suspicious lesions of the detected lesions [11]. Then, DL technology has now significantly advanced, increasing expectations for the likelihood of computer software relevant to tumor screening again. Deep learning (DL) is a type of neural network (NNs). This NN has an output layer, an input layer, and a hidden layer. DL can be a NN with a lot of hidden layers. In the past, DL had more achievements, i.e., incredible performance improvements, particularly in speech recognition and image classification [12]. Recently, DL has been utilized in various areas. As they can solve complicated issues, DNNs are now common in the healthcare field. However, decision-making by these methods was fundamentally a black-box procedure making it problematic for doctors to determine whether choices were dependable. The usage of explainable artificial intelligence (XAI) can be recommended as the key to this issue [13].

### 1.1. Related Works

Van der Velden et al. [14] presented an outline of explainable AI (XAI) utilized in DL-related medical image analysis. A structure of XAI criteria can be presented for classifying DL-related medical image analysis techniques. As per the structure and anatomical location, studies on the XAI mechanism in medical image analysis were categorized and surveyed. Esmaeili et al. [15] intend to assess the performance of selective DL methods on localizing cancer lesions and differentiating lesions from healthier areas in MRI contrasts. Despite an important correlation between lesion localization accuracy and classification, the familiar AI techniques inspected in this study categorize certain cancer brains dependent upon other non-related attributes. The outcomes advocate that the abovementioned AI methods can formulate an intuition for method interpretability and play a significant role in the performance assessment of DL methods.

In [16], a new automatic classification system by merging several DL methods was devised for identifying prostate cancer from MRI and ultrasound (US) imageries. To enrich the performance of the model, particularly on the MRI data, the fusion model can be advanced by integrating the optimal pretrained method as feature extractors with shallow ML techniques (e.g., K-NN, SVM, RF, and Adaboost). At last, the fusion model can be inspected by explainable AI to identify the fact why it finds samples as Malignant or Benign Stage in prostate tumors. Kobylińska et al. [17] modeled selective techniques from the XAI domain in the instance of methods implemented for assessing lung cancer risk in the screening process of lung cancer using low-dose CT. The usage of such methods offers a good understanding of differences and similarities among the three typically used methods in screening lung cancer they are LCART, BACH, and PLCOm2012.

In [18], an explainable AI (XAI) structure was devised in this study for presenting the local and global analysis of auxiliary identification of hepatitis while maintaining good predictive outcomes. Firstly, a public hepatitis classifier benchmark from UCI was utilized for testing the structure feasibility. Afterward, the transparent and black-box ML methods were used to predict the deterioration of hepatitis. Transparent methods like KNN, LR, and DT were selected. While the black-box method like the RF, XGBoost, and SVM were selected. Watson and Al Moubayed [19] devised a method agnostic explainability-related technique for the precise identification of adversarial instances on two datasets with various properties and complexity: chest X-ray (CXR) data and Electronic Health Record (EHR). In [20], the XAI tool can be applied to the breast cancer (BC) dataset and offers a graphical analysis. The medical implication and molecular processes behind circulating adiponectin, HOMA, leptin, and BC resistance were sightseen, and XAI techniques were utilized for constructing methods for the diagnosis of new BC biomarkers.

### 1.2. Paper Contributions

This study develops an Adaptive Aquila Optimizer with Explainable Artificial Intelligence Enabled Cancer Diagnosis (AAOXAI-CD) technique on Medical Imaging. The proposed AAOXAI-CD technique uses the Faster SqueezeNet model for feature vector generation. As well as the execution of hyperparameter tuning of the Faster SqueezeNet model done with the AAO algorithm. For cancer classification, the majority weighted voting ensemble model with three DL classifiers, namely recurrent neural network (RNN), gated recurrent unit (GRU), and bidirectional long short-term memory (BiLSTM). Furthermore, the AAOXAI-CD technique combines the XAI approach LIME for better understanding and explainability of the black-box method for accurate cancer detection. The simulation evaluation of the AAOXAI-CD technique is tested on medical cancer imaging databases.

## 2. Materials and Methods

**Figure 2:**
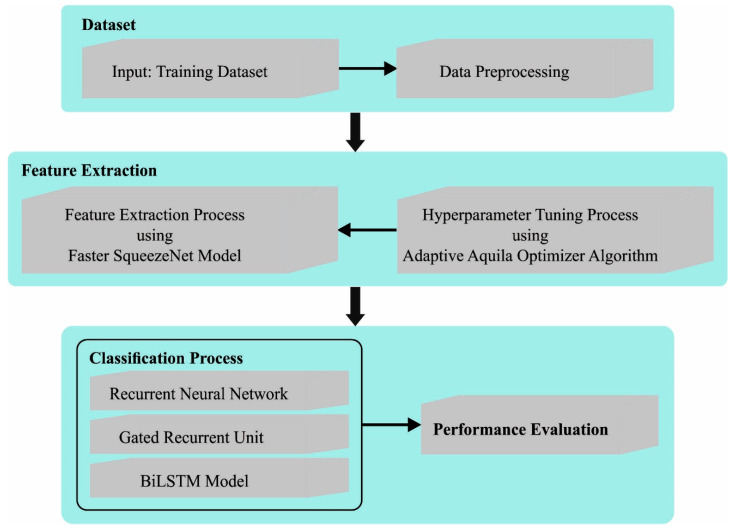
The overall flow of AAOXAI-CD approach.

In this article, we have developed an automated cancer diagnosis approach using the AAOXAI-CD approach on medical images. The proposed AAOXAI-CD system attained the effectual colorectal and osteosarcoma cancer classification process. It encompasses Faster SqueezeNet-based feature vector generation, AAO-based parameter tuning, ensemble classification, and XAI modeling. Figure 2 defines the overall flow of the AAOXAI-CD approach. The overall process involved in the proposed model is given in Algorithm 1.

**Algorithm 1**: Process Involved in AAOXAI-CD TechniqueStep 1: Input Dataset (Training Images)Step 2: Image Pre-ProcessingStep 3: Feature Extraction Using Faster SqueezeNet ModelStep 4: Parameter Tuning Process   Step 4.1: Initialize the Population and Its Parameters   Step 4.2: Calculate the Fitness Values   Step 4.3: Exploration Process and Exploitation Process   Step 4.4: Update the Fitness Values   Step 4.5: Obtain Best SolutionStep 5: Ensemble of Classifier (RNN, GRU, and Bi-LSTM)Step 6: Classification Output

### 2.1. Feature Extraction Using Faster SqueezeNet

**Figure 3:**
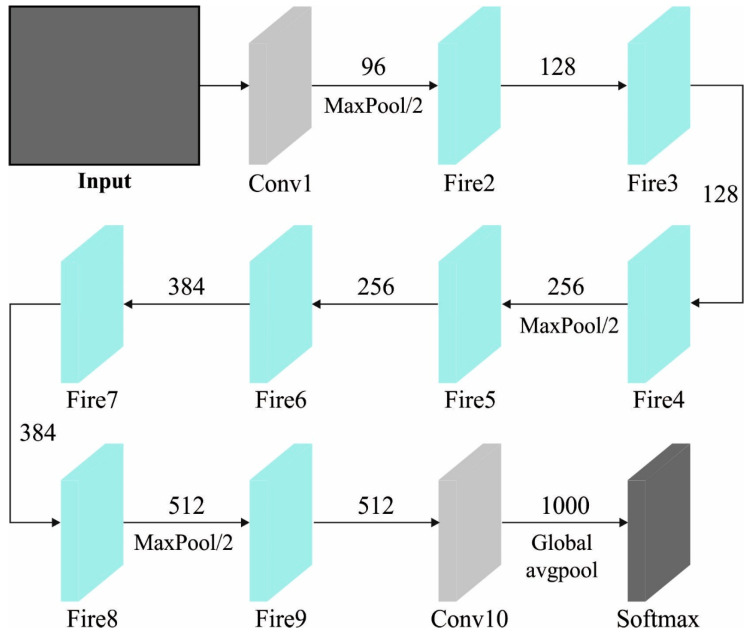
Architecture of Faster SqueezeNet.

Primarily, the AAOXAI-CD technique employed the Faster SqueezeNet method for feature vector generation. Fast SqueezeNet was proposed to enrich the real-time performance and accuracy of cancer classification [21]. We added BatchNorm and residual structure to prevent overfitting. Simultaneously, like DenseNet, concat is employed to interconnect dissimilar layers to increase the expressiveness of the first few layers in the network. Figure 3 represents the architecture of the Faster SqueezeNet method.

Fast SqueezeNet comprises a global average pooling layer, 1 BatchNorm layer, 3 block layers, and 4 convolutional layers. In the following ways, Fast SqueezeNet can be improved:

(1) To further enrich the information flow among layers DenseNet is imitated, and a distinct connection mode is devised. This covers a fire module and pooling layer, and lastly, 2 concat layers are interconnected to the following convolution layer.

The present layer receives each feature map of the previous layer, and we apply $x_{0},\ldots ,\;x_{l-1}$ as input; then, $x_{l}$ is expressed as

(1) $$x_{l}=H_{l}\left(\left[x_{0},x_{1},\ldots ,x_{l-1}\right]\right),$$

where $\left[x_{0},x_{1},\ldots ,x_{l-1}\right]$ represent the connection of feature graphs produced in layers 0, 1, …, l−1 and $H_{l}\left(\cdot \right)$ concatenated more than one input data. Now,  characterizes the max pooling layer, $x_{1}$ designates Fire layers, and $x_{l}$ indicates the concat layer.

Initially, the performance of the network is improved without excessively raising the number of network variables, and simultaneously, any two-layer network could directly transmit data.

(2) We learned from the ResNet structure and suggested constituent elements, which comprise a fire module and pooling layer, to ensure improved network convergence. Lastly, afterward, two layers were summed, and it was interconnected to the next convolution layers.

In ResNet, shortcut connection employs identity mapping that implies input of a convolutional stack will be added directly to the resultant of the convolutional stack. Formally, the underlying mapping can be represented as H (x), considering the stacked non-linear layer fits another mapping of F(x):=H(x)−x. The original mapping is rewritten into F(x)+x. F(x)+x is comprehended by the structure named shortcut connection in the encrypting process.

In this work, the hyperparameter tuning of the Faster SqueezeNet method occurs by employing the AAO algorithm. This abovementioned algorithm is based on the distinct hunting strategies of Aquila for different prey [22]. For faster-moving prey, the Aquila needs to obtain the prey in a precise and faster manner, where the global exploration capability of the model was reflected. The optimizer technique was characterized by mimicking 4 behaviors of Aquila hunting. Firstly, the population needs to arbitrarily generate in-between the lower bound (LB) and upper bound (UB) dependent upon the problem, as given in Equation (2). The approximate optimum solution at the time of the iteration can be defined as the optimum solution. The present set of candidate solutions X was made at random by using the following expression:

(2) $$X=\left[\begin{array}{lll} \chi_{1_{\text{’}}1} & \ldots & x_{1_{\text{’}}D}\\ \vdots & \ddots & \vdots \\ x_{n_{\text{’}}1} & \ldots & x_{n_{\text{’}}D} \end{array}\right]$$

(3) $$X_{i,j}=rand\times \left(UB_{j}-LB_{j}\right)+LB_{j},\;i=1,2,\;\ldots ,\;Nj=1,2,\;\ldots D$$

where n signifies the overall amount of candidate solutions, D indicates the dimensionality of problems, and $x_{n,\;D}$ represents the location of n-th solutions in d dimensional space. Rand denotes a randomly generated value, and $UB_{j}$ and $LB_{j}$ signify the j-th dimensional upper and lower boundary of the problem.

Initially, choose search spaces by hovering above in vertical bends. Aquila hovers above to identify the prey area and rapidly choose the better prey region as follows:

(4) $$X_{1}\left(t+1\right)=X_{besi}\left(t\right)\times \left(1-\frac{t}{T}\right)+\left(X_{M}\left(t\right)-X_{besi}\left(t\right)\right)\times rand$$

(5) $$X_{M}\left(t\right)=\frac{1}{N}\overset{N}{\underset{i=1}{\sum }}X_{i}\left(t\right),\forall j=1,2,\;\ldots ,\;D$$

where $X_{1}\left(t+1\right)$ symbolizes the location of the individual at t+1  time , $X_{besi}\left(t+1\right)$ signifies the present global optimum site at the t-th iteration, T and t symbolize the maximal amount of iterations and the present amount of iterations, correspondingly, X(t) represents the average location of the individual at the existing iteration, and Rand represents the randomly generated value within [0,1] in Gaussian distribution. The next strategy was a short gliding attack in isometric flight. Aquila flies over the targeted prey to prepare for assault while they find prey region from a higher altitude. This can be formulated as

(6) $$X_{2}\left(t+1\right)=X_{best}\left(t\right)\times levy\left(D\right)+X_{R}\left(t\right)+\left(y-x\right)\times rand$$

(7) $$levy\;\left(D\right)=s\times \frac{u\times \sigma }{|v{|}^{\frac{1}{\beta }}}$$

(8) $$\sigma =\left(\frac{\Gamma \left(1+\beta \right)\times \sin\left(\frac{\pi \beta }{2}\right)}{\Gamma \left(\frac{1+\beta }{2}\right)\times \beta \times 2^{\left(\frac{\beta -1}{2}\right)}}\right)$$

where $X_{2}\left(t+1\right)$ denotes the new solution for the following iteration of t, D means spatial dimensions, *levy (D)* denotes Lévy flight distribution functions, X(t) indicates the arbitrary location of Aquila in [1, N], s take the values of 1.5, y and χ presents the spiral situations in search region as follows:

(9) y=r× cos (θ)

(10) x=r× sin (θ)

(11) $$r=r_{1}+0.00565\times D_{1}$$

(12) $$\theta =-0.005\times D_{1}+\frac{3\times \pi }{2}$$

where $r_{1}$ takes the fixed index between 1 and 20, $D_{1}$ denotes the integers from 1 to the length of the search region. The third strategy was a slow-descent attack and low-flying. The Aquila locks onto a hunting target in the hunting region and, with attack ready, makes the initial attacks in the vertical descent, thereby testing prey response. These behaviors are given as follows:

(13) $$X_{3}\left(t+1\right)=\left(X_{besi}\left(t\right)-X_{M}\left(t\right)\right)\times \alpha -rand+\left(\left(UB-LB\right)\times rand+LB\right)\times \delta$$

where $X_{3}\left(t+1\right)$ denotes the solution of the following iteration of t, δ, and α denotes the mining adjustment parameter within (0,1), LB and UB represent the lower and upper boundaries of the issue. The fourth strategy was grabbing and walking prey. Once the Aquila approaches the prey, it starts to attack prey based on arbitrary movements of prey. These behaviors can be described as follows

(14) $$X_{4}\left(t+1\right)=QF\times X_{best}\left(t\right)-\left(G_{1}\times X\left(t\right)\times rand\right)-G_{2}\times levy\left(D\right)$$

(15) $$QF\left(t\right)=t^{\frac{2\times rand-1}{{\left(1-T\right)}^{2}}}$$

(16) $$G_{1}=2\times rand-1$$

(17) $$G_{1}=2\times \left(1-\frac{t}{T}\right)$$

where $X_{4}\left(t+1\right)$ denotes the new solution for the following iteration of t, QF represents the mass function leveraged for balancing the search process, and F∈(0,1) $G_{1}$ represents various strategies utilized by the Aquila for prey escape; $G_{2}$ signifies slope value from the initial location to the final location at the chase time of Aquila’s prey, which takes values from 2 to 0,⋅ Rand denotes the random number within [0,1] in Gaussian distribution; and T and t denotes the maximal amount of iterations and existing amount of iterations, correspondingly. Niche thought is from biology in which microhabitats represent roles or functions of the organization in a specific environment, and organizations with general features are named species. In the AAO algorithm, Niche thought is used, which applies a sharing model for comparing the distance among individuals in a habitat. A specific threshold was set to increase the fitness of an individual with the highest fitness, ensuring that the individual state is optimal. For an individual with the lowest fitness, a penalty was presented to make them update and further find the optimum value in another region to guarantee the diversity of the population at the iteration and attain the optimum solution. Here, the distance among individuals of the smallest habitat population was evaluated as follows:

(18) $$d_{ij}=\left|X_{i}-X_{j}\right|$$

The data exchange function among *Xi* and *Xj* individuals is given below

(19) $$sh\left(d_{ij}\right)=\{\begin{array}{l} 1-\frac{d_{i}i}{\rho },d_{ij}<\rho \\ 0,\;d_{ij}>\rho \end{array}$$

where ρ denotes the radius of data sharing in microhabitats and $d_{ij}<\rho$ guarantees that individuals live in the microhabitat environments. After sharing the data, the optimum adaptation can be adjusted in time, as follows.

(20) $$F_{i-}best=\frac{F_{i}}{sh},i=1,2,\;\ldots ,\;N$$

where $F_{i}$ means optimum adaptation after sharing, and $F_{j}$ denotes original adaptation.

The AAO method not only derived a fitness function from attaining superior classification performance as well describes positive values to symbolize the enhanced outcome of the candidate solutions. The reduction of classification error rates was treated as the fitness function.

(21) $$\begin{array}{cc} fitness\left(x_{i}\right) & =ClassifierErrorRate\left(x_{i}\right)\\ & =\frac{number\;of\;misclassified\;samples}{Total\;number\;of\;samples}\times 100 \end{array}$$

### 2.2. Ensemble Learning-Based Classification

In this work, the DL paradigm is integrated, and the best outcome is selected by the weighted voting method. Assumed the D base classification model and amount of classes as n for voting, predictive class $c_{k}$ of weighted voting for every instance as follows

(22) $$c_{k}=\underset{j}{\arg\;\max}\sum_{i=1}^{D}\left(\Delta_{\mathrm{ji}}\times w_{i}\right),$$

where $\Delta_{\mathrm{ji}}$ signifies binary parameter. As soon as ith base classification classifies the k instances into jth classes, then $\Delta_{\mathrm{ji}}=1$; or else, $\Delta_{\mathrm{ji}}=0$. $w_{i}$ represents the weight of ith base classification in the ensemble.

(23) $$\mathrm{Acc}=\frac{\sum_{k}\left\{1|c_{k}\mathrm{is}\;\mathrm{the}\;\mathrm{true}\;\mathrm{class}\;\mathrm{of}\;\mathrm{instance}\;k\right\}}{\mathrm{Size}\;\mathrm{of}\;\mathrm{test}\;\mathrm{instances}}\times 100\%.$$

#### 2.2.1. RNN Model

Initially, Elman recommended the recurrent unit as its essential block (1990). If they are used to exceedingly long sequences, the elementary RNN cell has common problems of expanding gradient and disappearing gradient [23]. It is a fact that the elementary RNN cell could not hold long-term dependence eventually. Hence it demonstrates that this cell has shortcomings. The backpropagated gradient tends to reduce once the sequence is particularly long, which prevents the effective updating of the weight. However, once the gradient is substantial, they might explode across a longer sequence, which renders the weight matrix unstable. The above two difficulties stem from the intractable nature of the gradient, which has made it more difficult for RNN cells to identify and be accountable for a long-term relationship. Equations (24) and (25) demonstrate the mathematical expression for RNN architecture.

(24) $$h_{t-1}=\sigma \left(P_{h}\times h_{t-1}+P_{x}\times x_{t}+B_{a}\right)$$

(25) $$y_{t}=\;\tan h\;\left(P_{o}\times h_{t}+B_{o}\right)$$

where $h_{t}$ denotes the hidden state, and it was the only type of memory in the RNN cell. $P_{h}$ and $P_{x}$ epitomize the weight matrix for the hidden state and $P_{o}$ bias vector for cell output correspondingly, $x_{t}$ and $y_{t}$ characterize the inputs and outputs of the cell at the *t* time step, correspondingly, $B_{a}$ and $B_{o}$ represent the bias vector for the hidden state and cell outputs, correspondingly.

The latter hidden state is conditioned on the hidden state of the previous time step and the existing inputs. The cellular feedback loop connects the current state to the succeeding one. This bond is crucial to consider prior data while adjusting the present cell state. In such cases, the hyperbolic tangent function, represented by Tanh, turned on the overt state, and the sigmoid function was applied, represented by, to turn on the latent state.

#### 2.2.2. GRU Model

The RNN is a kind of ANN model with a cyclic structure and is appropriate for data processing in sequence. The gradient is lost, and learning ability is greatly reduced once the time interval is large [24]. Hochreiter and Schmidhuber resolved these problems and developed the LSTM. The LSTM was extensively applied in time-series data, and its basic concept is that the cell state was interconnected as a conveyor belt. In that regard, the gradient propagates although distance among the states rises. In LSTM cells, the cell state can be controlled by using three gating functions forget, input, and output gates. In 2014, the GRU was developed as a network that enhanced the learning accuracy of LSTM by adjusting the LSTM model. Different from LSTM, the GRU has a fast-learning speed and is encompassed two gating functions. Furthermore, parameters are smaller than LSTM since the hidden and cell states are incorporated into a single hidden state. Accordingly, the GRU shows outstanding performance for long-term dependency in time-series data processing and takes lesser computational time when compared to the LSTM. The GRU equations to determine the hidden state are shown below:

(26) $$r_{t}=\sigma \left(W_{r}x_{t}+U_{r}h_{t-1}+b_{r}\right)$$

(27) $$z_{t}=\sigma \left(W_{z}x_{t}+U_{z}h_{t-1}+b_{z}\right)$$

(28) $$h_{t}=\left(1-z_{t}\right)⊙h_{t-1}+z_{t}⊙\tan h\;\left(W_{h}x_{t}+U_{h}\left(r_{t}E⊙h_{t-1}\right)+b_{h}\right)$$

From the expression, $r_{t}$ denotes the reset gate and $z_{t}$ indicates the update gate at time t. $x_{t}$ represents input value at t time, W and U indicate weights, and b refers to bias. $h_{t}$ denotes the hidden state at time t. ⊙ shows the component-wise (Hadamard) multiplication.

#### 2.2.3. BiLSTM Model

RNN has the structural feature of the node connected in a loop, making them appropriate for data processing; however, it is frequently confronted with the problem of vanishing gradient [25]. The GRU and long and short-term memory (LSTM) improved on RNN by adding several threshold gates to mitigate gradient vanishing problems and enhance classification accuracy. Meanwhile, the LSTM method has a memory unit that prevents the network from facing gradient vanishing problems.

The LSTM could enhance the deficiencies of RNN; generally, the resultant of the present time was relevant to the state information of the past time, as well as state information of future time. The Bi-LSTM network was established concerning the problem that was integrating historical and future data by interconnecting two LSTMs. The architecture of the BiLSTM network comprises the back-to-forth and front-to-back LSTM layers. The forward and backward layers calculate the input dataset, and lastly, the architecture of two layers is integrated to obtain the output of the BiLSTM network as follows:

(29) $$o_{t}=g\left(\omega_{1}i_{t}+\omega_{2}0_{t-1}\right)\;o_{t}^{’}=g\left(\omega_{3}i_{t}+\omega_{5}0_{t-1}^{’}\right),\;y_{t}=f(\omega_{4}0_{t}+\omega_{6}0_{t})$$

In Equation (29), ω denotes weighted parameters in the BiLSTM network, $i_{t}$ shows input at t time , $0_{t}$ indicates the results of the forward hidden layer at  t time, $0_{t}^{’}$ represents the output of the backward hidden layer at t time and $y_{t}$ represents the last resultant of the network.

### 2.3. Modeling of XAI Using LIMA Approach

The AAOXAI-CD technique combines the XAI approach LIME for a better understanding and explainability of the black-box method for accurate cancer detection [26]. Local interpretable model-agnostic explanation (LIME) describes various ML approaches for regression prediction, using the featured value change of the data sample to transform the featured values into the contribution of the predictor. The explainer gives a local interpretation of the data samples. For example, the interpretable model in LIME often uses linear regression (LR) or decision trees (DTs) and are trained by the smaller perturbation (removing specific words, hiding part of the image, and adding random noise) in the model. The quality of these models seems to be increasing and was used to resolve the best part of the business victimization dataset. Similarly, there were persistent tradeoffs between model accuracy and interpretability. Generally, the performance can be improved and enhanced by applying sophisticated techniques such as call trees, random forest, material, boosting, and SVM, which are “blackbox” techniques. The LIME provides a clear explanation of the problems with the blackbox classifiers. The LIME is a way of understanding an ML BlackBox method by perturbing the input dataset and seeing how prediction changes. The LIME is used for any ML black-box models. The fundamental steps are shown as follows:

A TabularExplainer is initialized by the data used for the data training about the features and various class names.

In the class explain_instance, a technique called explain_instance accepts the reference to the instance where the explanation is essential, plus the number of features to be added in the explanation and the trained model’s prediction technique.

## 3. Results and Discussion

**Figure 4:**
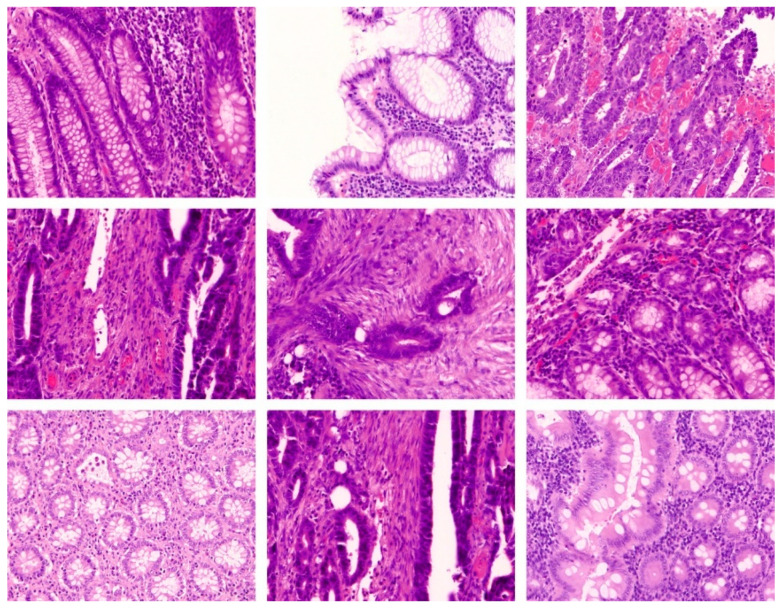
Sample Images of Colorectal Cancer.

**Figure 5:**
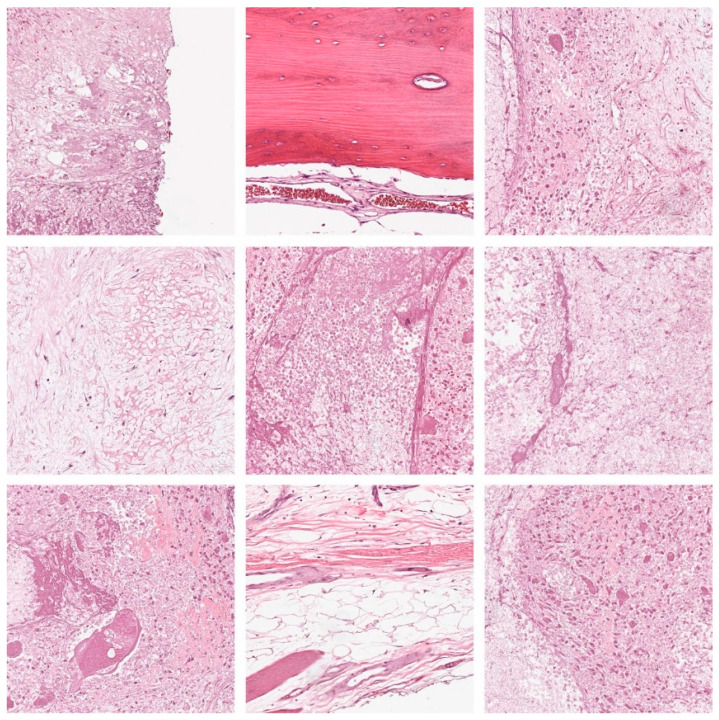
Sample images of osteosarcoma.

The proposed model is simulated using Python 3.6.5 tool on PC i5-8600k, GeForce 1050 Ti 4 GB, 16 GB RAM, 250 GB SSD, and 1 TB HDD. The parameter settings are given as follows: learning rate: 0.01, dropout: 0.5, batch size: 5, epoch count: 50, and activation: ReLU. In this section, the simulation values of the AAOXAI-CD technique can be tested utilizing dual datasets: the colorectal cancer dataset (dataset 1) and the osteosarcoma dataset (dataset 2). Figure 4 defines the sample images of Colorectal Cancer. For experimental validation, 70:30 and 80:20 of the training set (TRS) and testing set (TSS) is used. Dataset 1 (Warwick-QU dataset) [27] comprises 165 images with 91 malignant tumors and 74 benign tumor images. The data were collected using the Zeiss MIRAX MIDI Scanner by implementing an image data weight range of 1.187 kilobytes, 716 kilobytes, and an image data resolution range of 567 × 430 pixels to 775 × 522 pixels with all pixels having a distance of 0.6 µm from the actual distance. Next, dataset 2 [28] contains 1144 images under 3 classes. It covers 536 images under Non-Tumor (NT) class, 345 images under viable tumor (VT), and 263 images under non-Viable Tumor (NVT). Figure 5 defines the sample images of osteosarcoma.

**Figure 6:**
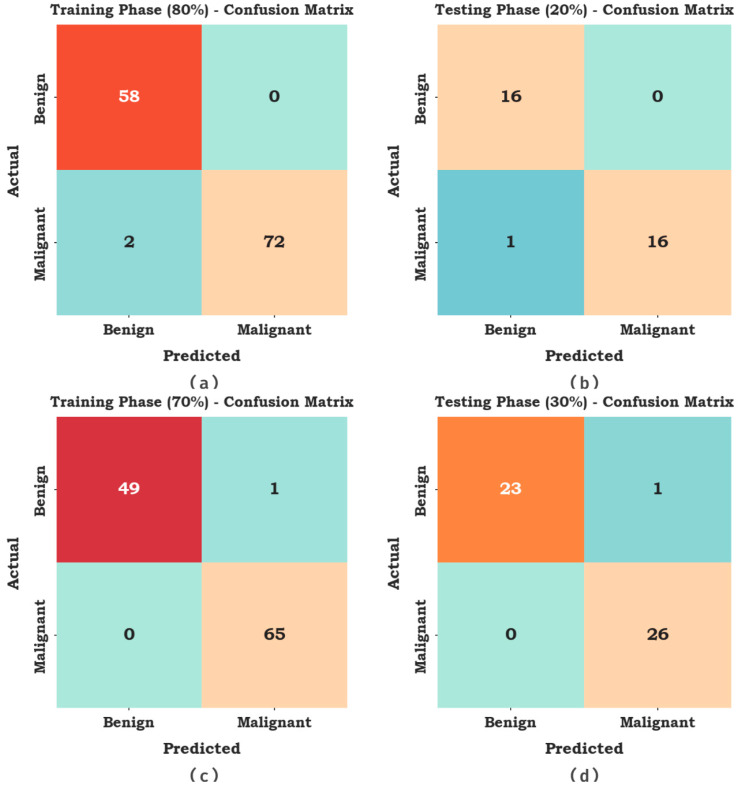
Confusion matrices of the AAOXAI-CD system on dataset-1 (**a**,**b**) TRS/TSS of 80:20 and (**c**,**d**) TRS/TSS of 70:30.

In Figure 6, the cancer classifier outcomes of the AAOXAI-CD method in terms of classification performance under dataset-1. The outcomes demonstrate that the AAOXAI-CD system has identified benign and malignant samples.

In Table 1, the overall classifier results of the AAOXAI-CD method on dataset-1. The results demonstrate that the AAOXAI-CD method has identified benign and malignant samples. For instance, with 80% of TRS, the AAOXAI-CD technique reaches an average $accu_{y}$ of 98.65%, $prec_{n}$ of 98.33%, $reca_{l}$ of 98.65%, $spec_{y}$ of 98.65%, $F_{score}$ of 98.47%, and MCC of 96.98%. Meanwhile, with 20% of TSS, the AAOXAI-CD system reaches an average $accu_{y}$ of 97.06%, $prec_{n}$ of 97.06%, $reca_{l}$ of 97.06%, $spec_{y}$ of 97.06%, $F_{score}$ of 96.97%, and MCC of 94.12%. Furthermore, with 70% of TRS, the AAOXAI-CD algorithm reaches an average $accu_{y}$ of 99%, $prec_{n}$ of 99.24%, $reca_{l}$ of 99%, $spec_{y}$ of 99%, $F_{score}$ of 99.11%, and MCC of 98.24%.

**Figure 8:**
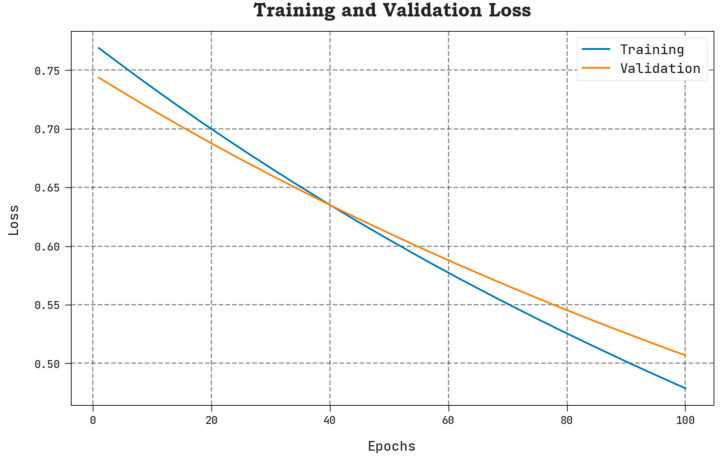
TLOS and VLOS analysis of AAOXAI-CD approach on dataset-1.

The TLOS and VLOS of the AAOXAI-CD model on dataset-1 are defined in Figure 8. The figure inferred that the AAOXAI-CD approach has superior performance with minimal values of TLOS and VLOS. Notably, the AAOXAI-CD model has minimal VLOS outcomes.

**Figure 9:**
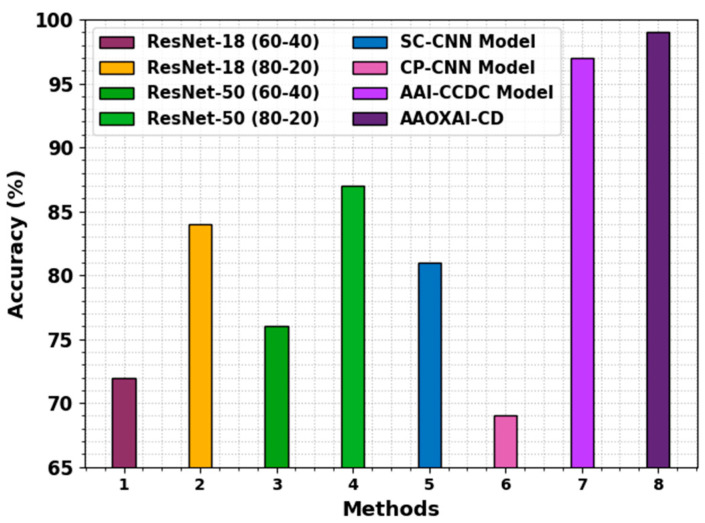
Comparative analysis of the AAOXAI-CD approach on dataset-1.

In Table 2 and Figure 9, the comparative interpretation of the AAOXAI-CD system with recent methods on dataset-1 [29,30,31]. The figures represented that the ResNet-18(60–40), ResNet-50 (60–40), and CP-CNN models resulted in the least performance. Although the AAI-CCDC technique results in moderately improved outcomes, the AAOXAI-CD technique accomplishes maximum performance with $prec_{n}$ of 99.24%, $reca_{l}$ of 99%, and $accu_{y}$ of 99%.

**Figure 10:**
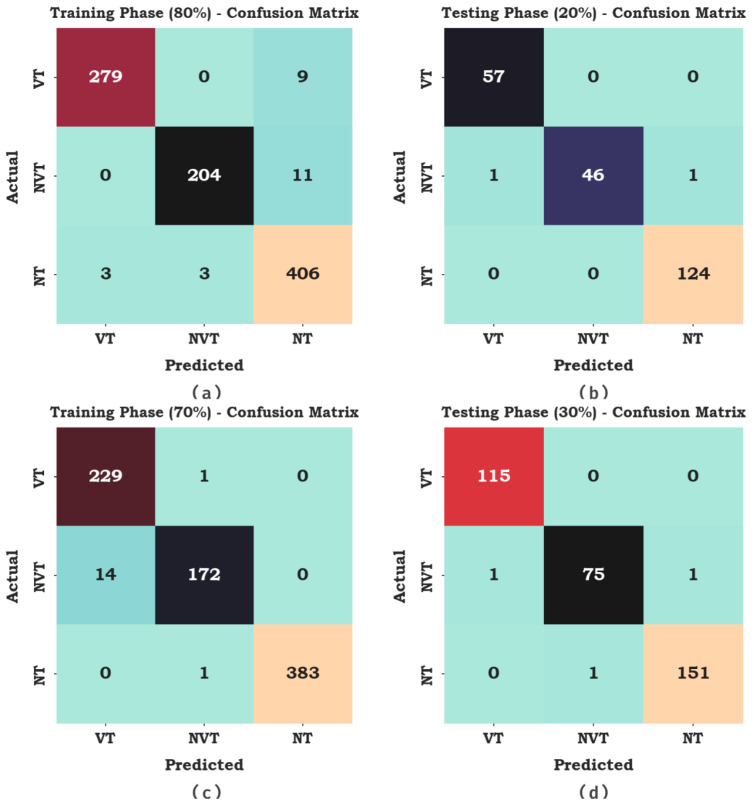
Confusion matrices of AAOXAI-CD system on dataset-2 (**a**,**b**) TRS/TSS of 80:20 and (**c**,**d**) TRS/TSS of 70:30.

In Figure 10, the cancer classification outcomes of the AAOXAI-CD system in terms of classification performance under dataset-2. The results demonstrate that the AAOXAI-CD technique has identified benign and malignant samples.

In Table 3, the overall classifier results of the AAOXAI-CD system on dataset-2. The results demonstrate that the AAOXAI-CD method has identified benign and malignant samples. For instance, with 80% of TRS, the AAOXAI-CD technique reaches an average $accu_{y}$ of 98.11%, $prec_{n}$ of 97.60%, $reca_{l}$ of 96.77%, $spec_{y}$ of 98.37%, $F_{score}$ of 97.16%, and MCC of 95.66%. Meanwhile, with 20% of TSS, the AAOXAI-CD algorithm reaches an average $accu_{y}$ of 99.42%, $prec_{n}$ of 99.16%, $reca_{l}$ of 98.61%, $spec_{y}$ of 99.49%, $F_{score}$ of 98.87%, and MCC of 98.44%. Furthermore, with 70% of TRS, the AAOXAI-CD technique reaches an average $accu_{y}$ of 98.67%, $prec_{n}$ of 97.70%, $reca_{l}$ of 97.26%, $spec_{y}$ of 99.07%, $F_{score}$ of 97.42%, and MCC of 96.56%.

**Figure 12:**
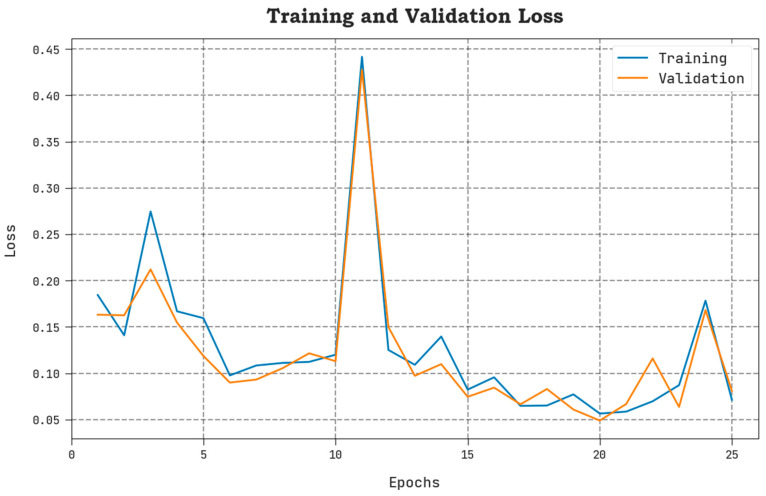
TLOS and VLOS analysis of AAOXAI-CD method on dataset-2.

The TLOS and VLOS of the AAOXAI-CD model on dataset-2 are defined in Figure 12. The figure inferred the AAOXAI-CD system has better outcomes having minimal values of TLOS and VLOS. Visibly the AAOXAI-CD model has minimal VLOS outcomes.

**Figure 13:**
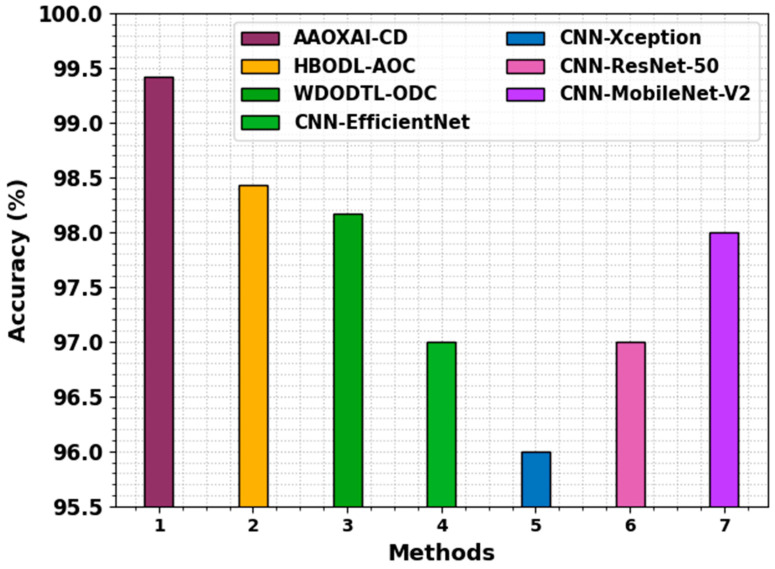
Comparative analysis of the AAOXAI-CD approach on dataset-2.

Table 4 and Figure 13 show a brief study of the AAOXAI-CD method with the recent method on dataset-2 [32,33]. The experimental values represented that the CNN-Xception, CNN-EfficientNet, CNN-ResNet-50, and CNN-MobileNet-V2 models resulted in the least performance. Although the WDODTL-ODC and HBODL-AOC techniques result in moderately improved outcomes, the AAOXAI-CD technique accomplishes maximum performance with of $prec_{n}$ 99.05%, of $reca_{l}$ 98.91%, and $accu_{y}$ of 99.42%.

From the above-mentioned results, it is assured that the proposed model achieves effectual classification performance over other DL models. The enhanced performance of the proposed model is due to the inclusion of AAO-based hyperparameter tuning and ensemble classification processes. In addition, the use of LIME helps to build an effective predictive modeling technique in cancer diagnosis. Without transparency, it is hard to gain the trust of healthcare professionals and employ predictive approaches in their daily operations. XAI has received considerable interest in recent times. It enables the clients to generate instances and comprehend how the classification model accomplishes the results. Healthcare institutions are keenly designing predictive models for supporting operations. The XAI can be combined to improve the transparency of healthcare predictive modeling. The interactions between healthcare professionals and the AI system are important for transferring knowledge and adopting models in healthcare operations.

## 4. Conclusions

In this study, we have developed an automated cancer diagnosis method using the AAOXAI-CD technique on medical images. The proposed AAOXAI-CD system attained the effectual colorectal and osteosarcoma cancer classification process. Primarily, the AAOXAI-CD technique utilized the Faster SqueezeNet model for feature vector generation. Moreover, the hyperparameter tuning of the Faster SqueezeNet model takes place with the AAO algorithm. For cancer classification, the majority-weighted voting ensemble model with three DL classifiers, namely RNN, GRU, and BiLSTM. Furthermore, the AAOXAI-CD technique combines the XAI approach LIME for better understanding and explainability of the black-box method for accurate cancer detection. The experimental evaluation of the AAOXAI-CD approach was tested on medical cancer imaging databases, and the outcomes ensured the promising outcome of the AAOXAI-CD method over other recent methods. In the future, a feature fusion-based classification model can be designed to boost the performance of the AAOXAI-CD technique.

## Figures and Tables

**Figure 7:**
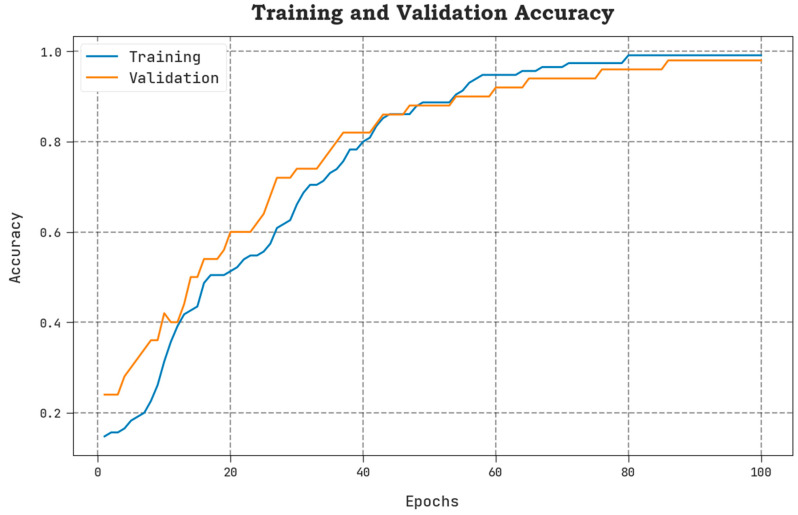
TACY and VACY analysis of the AAOXAI-CD approach on dataset-1.

**Figure 11:**
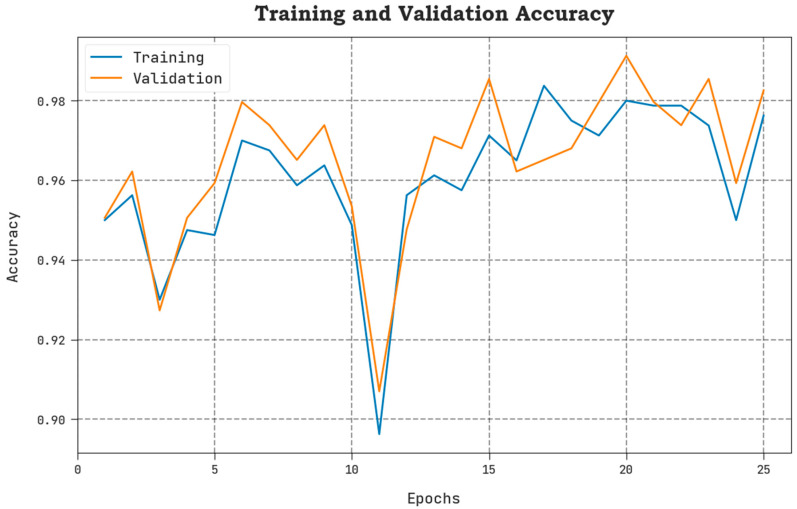
TACY and VACY analysis of AAOXAI-CD approach on dataset-2.

**Table 1:** Classifier outcome of the AAOXAI-CD approach on dataset-1.

Classes	$Accu_{y}$	$Prec_{n}$	$Reca_{l}$	$Spec_{y}$	$F_{score}$	MCC
Training Phase (80%)						
Benign	100.00	96.67	100.00	97.30	98.31	96.98
Malignant	97.30	100.00	97.30	100.00	98.63	96.98
Average	98.65	98.33	98.65	98.65	98.47	96.98
Testing Phase (20%)						
Benign	100.00	94.12	100.00	94.12	96.97	94.12
Malignant	94.12	100.00	94.12	100.00	96.97	94.12
Average	97.06	97.06	97.06	97.06	96.97	94.12
Classes	Accuracy	Precision	Recall	Specificity	F-Score	MCC
Training Phase (70%)						
Benign	98.00	100.00	98.00	100.00	98.99	98.24
Malignant	100.00	98.48	100.00	98.00	99.24	98.24
Average	99.00	99.24	99.00	99.00	99.11	98.24
Testing Phase (30%)						
Benign	95.83	100.00	95.83	100.00	97.87	96.06
Malignant	100.00	96.30	100.00	95.83	98.11	96.06
Average	97.92	98.15	97.92	97.92	97.99	96.06

The TACY and VACY of the AAOXAI-CD model on dataset-1 are defined in Figure 7. The figure exhibited that the AAOXAI-CD method has improvised performance with augmented values of TACY and VACY. Visibly, the AAOXAI-CD model has maximum TACY outcomes.

**Table 2:** Analysis outcome of AAOXAI-CD method with other systems on dataset-1.

Methods	Precision	Recall	Accuracy
ResNet-18 (60–40)	82.00	63.00	72.00
ResNet-18 (80–20)	86.00	82.00	84.00
ResNet-50 (60–40)	91.00	59.00	76.00
ResNet-50 (80–20)	82.00	92.00	87.00
SC-CNN Model	80.00	82.00	81.00
CP-CNN Model	71.00	68.00	69.00
AAI-CCDC Model	96.00	98.00	97.00
AAOXAI-CD	99.24	99.00	99.00

**Table 3:** Classifier outcome of AAOXAI-CD approach on dataset-2.

Classes	$Accu_{y}$	$Prec_{n}$	$Reca_{l}$	$Spec_{y}$	$F_{score}$	MCC
Training Phase (80%)						
VT	98.69	98.94	96.88	99.52	97.89	96.95
NVT	98.47	98.55	94.88	99.57	96.68	95.72
NT	97.16	95.31	98.54	96.02	96.90	94.32
Average	98.11	97.60	96.77	98.37	97.16	95.66
Testing Phase (20%)						
VT	99.56	98.28	100.00	99.42	99.13	98.85
NVT	99.13	100.00	95.83	100.00	97.87	97.36
NT	99.56	99.20	100.00	99.05	99.60	99.12
Average	99.42	99.16	98.61	99.49	98.87	98.44
Classes	$Accu_{y}$	$Prec_{n}$	$Reca_{l}$	$Spec_{y}$	$F_{score}$	MCC
Training Phase (70%)						
VT	98.12	94.24	99.57	97.54	96.83	95.57
NVT	98.00	98.85	92.47	99.67	95.56	94.35
NT	99.88	100.00	99.74	100.00	99.87	99.75
Average	98.67	97.70	97.26	99.07	97.42	96.56
Testing Phase (30%)						
VT	99.71	99.14	100.00	99.56	99.57	99.35
NVT	99.13	98.68	97.40	99.63	98.04	97.48
NT	99.42	99.34	99.34	99.48	99.34	98.82
Average	99.42	99.05	98.91	99.56	98.98	98.55

The TACY and VACY of the AAOXAI-CD model on dataset-2 are defined in Figure 11. The figure highlighted that the AAOXAI-CD method has performance with increased values of TACY and VACY. Remarkably, the AAOXAI-CD model has higher TACY outcomes.

**Table 4:** Comparative analysis of AAOXAI-CD approach with other systems on dataset-2.

Methods	Precision	Recall	Accuracy
AAOXAI-CD	99.05	98.91	99.42
HBODL-AOC	98.94	98.12	98.43
WDODTL-ODC	98.76	97.65	98.17
CNN-EfficientNet	97.00	97.00	97.00
CNN-Xception	94.00	96.00	96.00
CNN-ResNet-50	98.00	94.00	97.00
CNN-MobileNet-V2	98.00	98.00	98.00

## Data Availability

Data sharing is not applicable to this article as no datasets were generated during the current study.
